# Data on polyphenols and biological activity analyses of an Andean tomato collection and their relationships with tomato traits and geographical origin

**DOI:** 10.1016/j.dib.2016.04.005

**Published:** 2016-04-08

**Authors:** Romina D. Di Paola Naranjo, Santiago Otaiza, Alejandra C. Saragusti, Veronica Baroni, A.V. Carranza, Iris E. Peralta, Estela M. Valle, Fernando Carrari, Ramón Asis

**Affiliations:** a Facultad de Ciencias Químicas – CIBICI, Universidad Nacional de Córdoba – CONICET, Ciudad Universitaria, 5000 Córdoba, Argentina; b SECyT – ISIDSA/ICYTAC, Universidad Nacional de Córdoba – CONICET, Ciudad Universitaria, 5000 Córdoba, Argentina; c Facultad de Ciencias Agrarias, Universidad Nacional de Cuyo y CCT CONICET Mendoza, Mendoza, Argentina; d Instituto de Biología Molecular de Rosario, CONICET, Universidad Nacional de Rosario, Rosario, Argentina; e Instituto de Biotecnología, INTA Castelar, Argentina

## Abstract

Data provide information about a tomato collection composed of accessions from the Andean Valley, commercial accessions and wild species. Antioxidant metabolites were measured in mature fruits of this collection, and their biological activities were assessed by both in vitro and in vivo methods. In this work, the parameters used to identify and quantify polyphenols compounds in tomato fruit by liquid chromatography coupled to diode array detector and quadrupole time of flight mass spectrometer are described. Moreover, data supporting a procedure to characterize the properties of tomato fruits to revert death by thermal stress in *Caenorhabditis elegans* are explained in detail. Lastly, principal component analysis and hierarchical cluster analysis of metabolites composition, antioxidant activities (in vivo and in vitro), tomato traits and geographical origin of the tomatoes collection are shown. The data presented here are related to the research article entitled “Hydrophilic antioxidants from Andean Tomato Landraces assessed by their bioactivities in vitro and in vivo” [1].

## Specifications Table

**Table:** 

Subject area	*Biology*
More specific subject area	*Functional food and food composition*
Type of data	*Tables and figures*
How data was acquired	*Data of LC-DAD-MS/MS were acquired using an Agilent Technologies 1200 Series equipment connected to both Agilent G1315 C Starlight DAD and mass spectrometer (micrOTOF-Q11 Series, Bruker). Data of Caenorhabditis elegans (C. elegans) survival was acquired from a NIKON SMZ 645 Stereomicroscope*
Data format	*Raw, analyzed*
Experimental factors	*Hydrophilic antioxidants were extracted from tomato fruit with 75% (v/v) aqueous methanol*
Experimental features	*Tomato polyphenols were measured by LC-DAD-MS. Biological activity of fruit extracts was evaluated in terms of their capacity to confer tolerance to thermal stress in a C. elegans model. Principal component analysis and hierarchical cluster analysis were performed in order to find relationships between phenolic composition/in vivo and in vitro activities vs. tomato traits and geographical origin*
Data source location	*Estación experimental La Consulta, INTA, Mendoza and CIBICI, Facultad de Ciencias Químicas, Universidad Nacional de Córdoba, Córdoba, Argentina.*
Data accessibility	Data are available in this article

## Value of the data

• Data provide information on the method to identify and quantify polyphenols in tomato fruits by LC-DAD-QTOF.
• Data support the procedure in a *C. elegans* model to characterize the properties of tomato fruits to revert mortal stresses in living organisms.
• Data show relationships between antioxidant composition vs. tomato traits and antioxidant composition vs. geographical origins of tomato fruits.
• Data show relationships between antioxidant activities (in vivo and in vitro) vs. tomato traits and antioxidant activities vs. geographical origins of tomato fruits.

## 1 Data

**Fig. 1:**
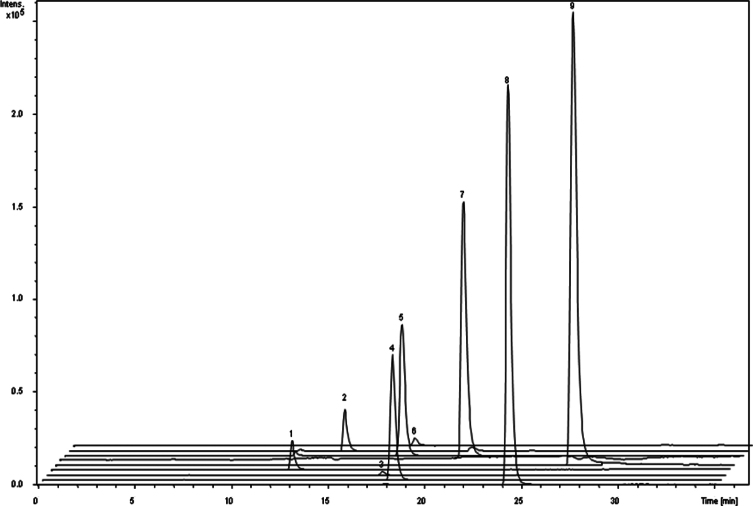
Extracted ion chromatogram of phenolic compounds standards. Peaks: (1) chlorogenic acid, (2) caffeic acid, (3) p-coumaric acid, (4) naringin, (5) ferulic acid, (6) rutin, (7) myricetin, (8) naringenin, (9) kaempferol.

**Fig. 2:**
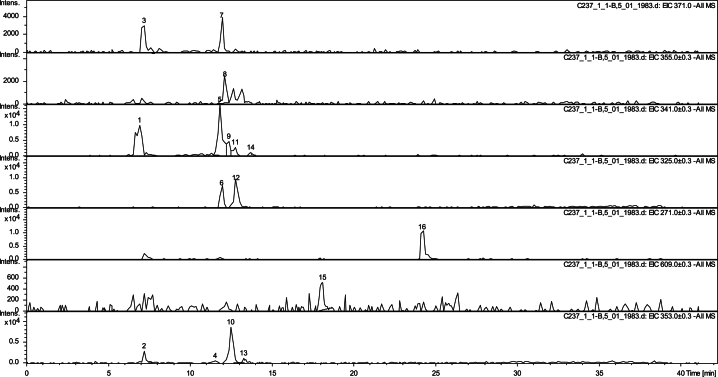
Extracted ion chromatogram of phenolic compounds identified in GPEA tomato accession. Peaks numbers designate identified compounds: (1) caffeic acid-O-hexose I, (2) chlorogenic acid isomer, (3) caffeoyl hexaric acid, (4) neochlorogenic acid, (5) caffeic acid-O-hexose II, (6) coumaric acid-O-hexose I, (7) trihydroxy cinnamoylquinic acid, (8) ferulic acid-O-hexoside, (9) caffeic acid-O-hexose III, (10) chlorogenic acid, (11) caffeic acid-O-hexose IV, (12) coumaric acid-O-hexose II, (13) cryptochlorogenic acid, (14) caffeic acid-O-hexose V, (15) rutin, (16) naringenin-chalcone.

**Fig. 3:**
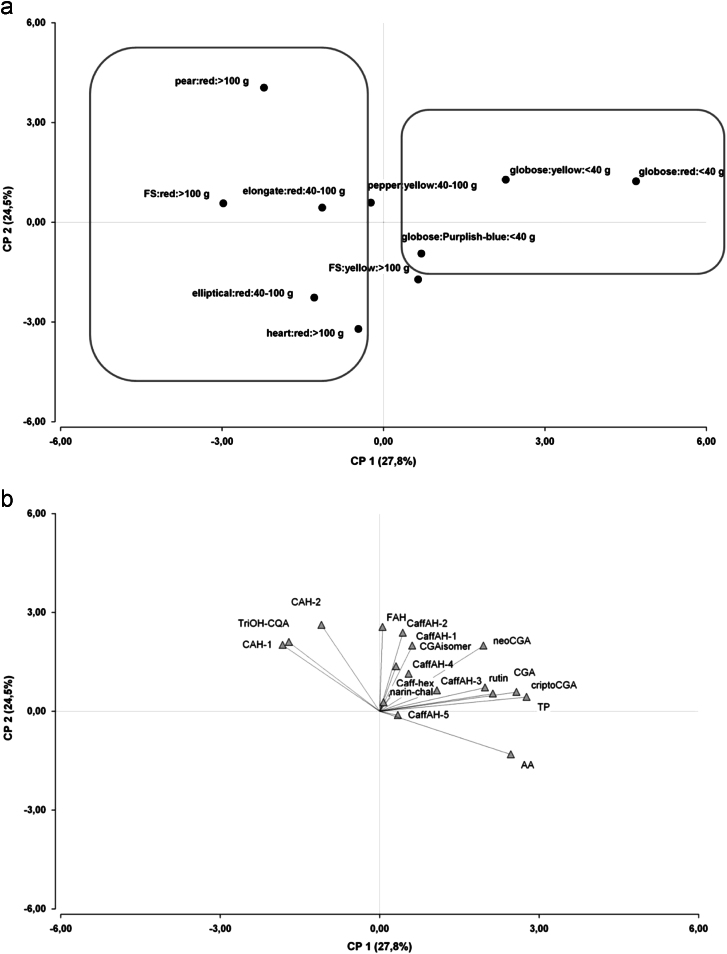
Principal component analysis of antioxidant compounds and fruit traits of tomato accessions. (a) Biplot graphic of fruit traits variables: color, size and shape. Accessions grouped by size were defined within the square. (b) Biplot graphic of hydrophilic antioxidant metabolites. AA means ascorbic acid and TP means total polyphenols and their quantification was described in the related research article [1].

**Fig. 4:**
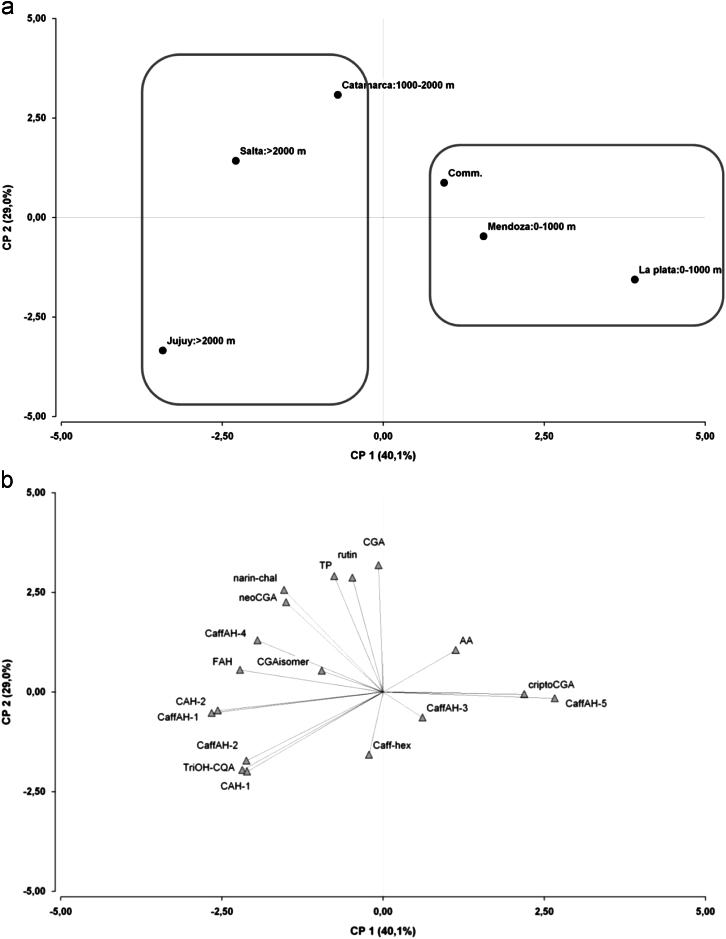
Principal component analysis of antioxidant compounds, geographical origin and altitude (meters above sea level) of tomato accessions. (a) Biplot graphic of locations and their altitude. Comm: commercial accessions with unknown geographical origin and altitude. Tomato accessions grouped by location are defined within the square. (b) Biplot graphic of hydrophilic antioxidant metabolites variables. AA means ascorbic acid and TP means total polyphenols and their quantification was described in the related research article [1].

**Fig. 5:**
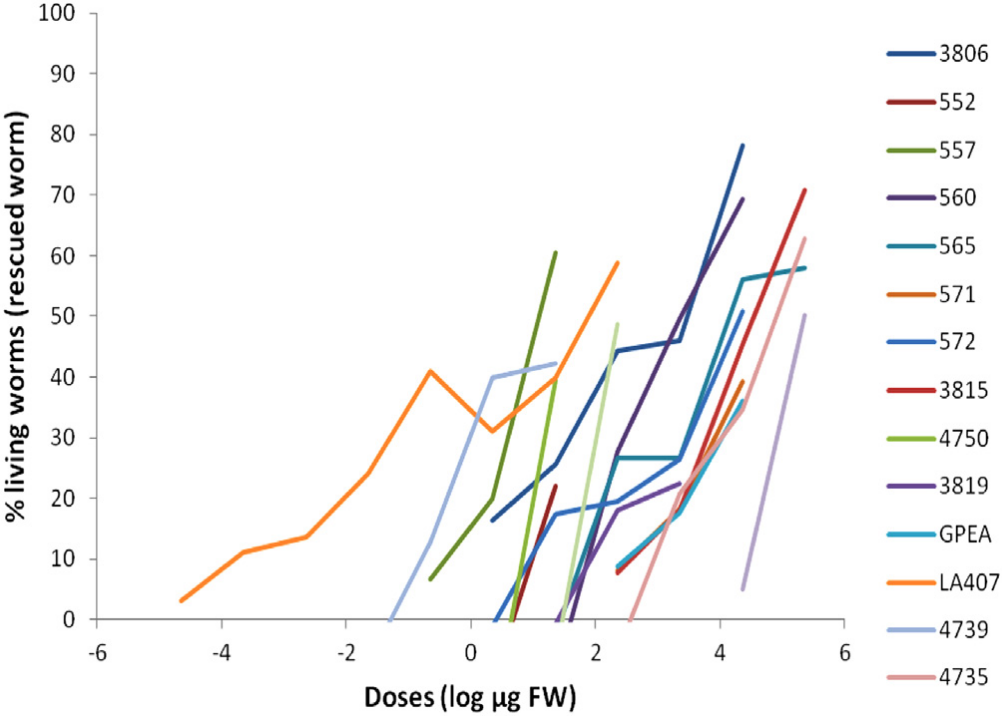
Doses–response curves of worms exposed to thermal stress, which were previously incubated with serial dilutions of extracts from tomato accessions. log µg FW: logarithm of micrograms of tomato (fresh weight).

**Fig. 6:**
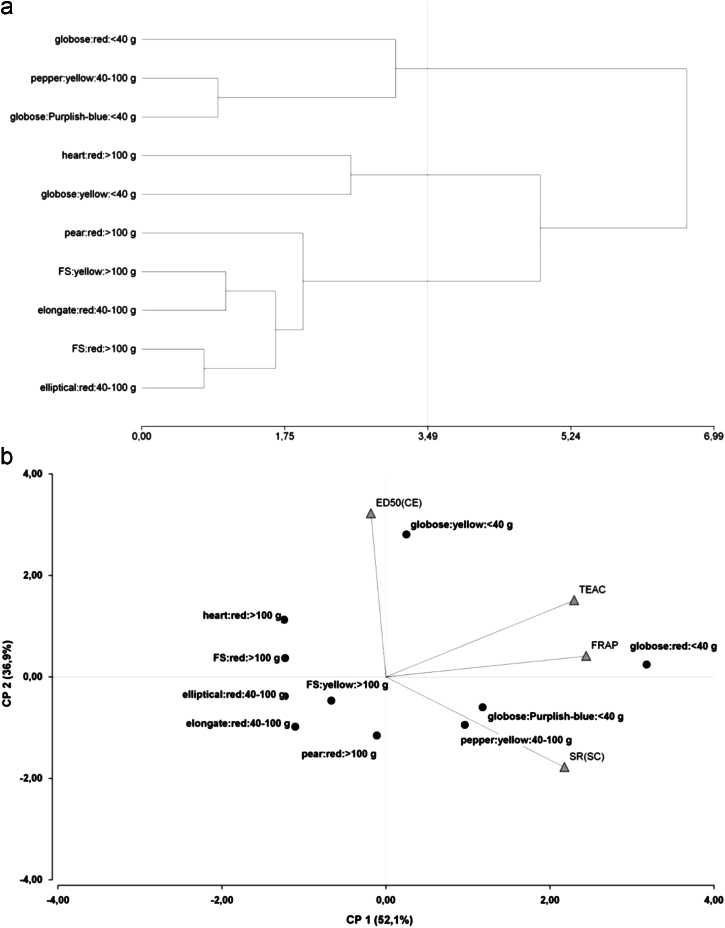
Hierarchical cluster analysis and principal component analysis of antioxidant activities and fruit traits of tomato accessions. (a) Complete linkage clustering method of fruit traits variables: shape, color and size. (b) Biplot graphic of traits and antioxidant activities variables. Trolox equivalent antioxidant capacity (TEAC) and ferric reducing ability of plasma (FRAP) represented the in vitro activities, and described in the related research article. ED50 (CE) means thermotolerance activity in *C. elegans* model and SR (SC) means the survival rate in *S. cerevisiae* model, both described in the related research article [1].

**Fig. 7:**
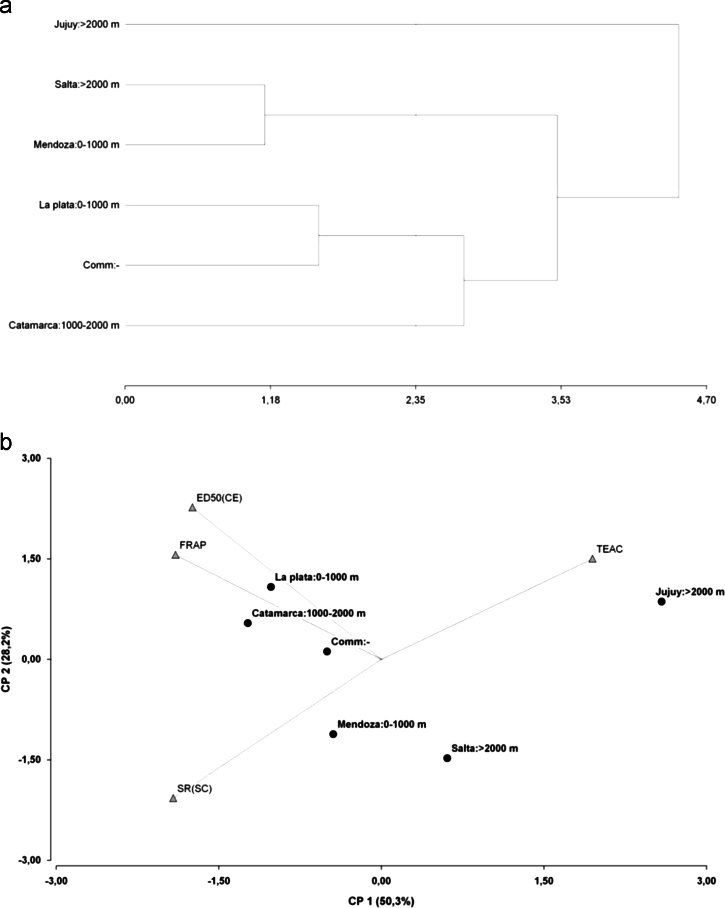
Hierarchical cluster analysis and principal component analysis (PCA) of antioxidant activities and geographical origin and altitude of tomato accessions. (a) Complete linkage clustering method of fruit geographical origin and altitude. (b) Biplot graphic of locations and altitude and antioxidant activities variables. Comm: commercial accession with unknown geographical origin and altitude. Trolox equivalent antioxidant capacity (TEAC) and ferric reducing ability of plasma (FRAP) represented the in vitro activities, and described in the related research article. ED50 (CE) means thermotolerance activity in *C. elegans* model, and SR (SC) means the survival rate in *S. cerevisiae* model, both described in the related research article [1].

Tomato accessions collected from Andean valleys, commercial varieties and wild species were characterized according to their fruit traits and geographical origin (Table 1). Fruit polyphenols levels were measured by LC-DAD-MS (Table 2, Figs. 1 and 2). Relationships between polyphenols composition, fruit traits and geographical origin were analyzed (Fig. 3, Fig. 4). The capacity of fruit extract to confer tolerance to thermal stress in a *Caenorhabditis elegans* model was evaluated (Fig. 5). Relationships between in vitro and in vivo antioxidant activities and fruit traits and geographical origin were analyzed (Fig. 6, Fig. 7).

## 2 Experimental design, materials and methods

Sixteen tomato accessions composed of tomatoes collected from Andean valleys, commercial tomatoes and wild species (Table 1) were obtained from the Germplasm Bank of INTA La Consulta Agriculture Experimental Station. Fruit polyphenols composition was measured by LC-DAD-MS (Table 2, Fig. 1, Fig. 2). Relationships between polyphenols composition and fruit traits and geographical origin were analyzed (Fig. 3, Fig. 4). The capacity of fruit extract to confer tolerance to thermal stress in a *C. elegans* model was evaluated (Fig. 5). Relationships between in vitro and in vivo antioxidant activities and fruit traits and geographical origin were analyzed (Fig. 6, Fig. 7).

### 2.1 Tomato collection

Table 1 compiles the tomato species, accessions numbers, geographical origin, fruit traits, and uses of 16 tomato accessions. At ripening stage defined by color and firmness, three fruits per plant were harvested from three individual plants, around 60 and 65 days after anthesis, and were immediately frozen with liquid nitrogen and kept in polyethylene tubes at −80 °C until use.

### 2.2 Polyphenols extraction

The obtention of tomato hydrophylics extracts is explained in detail in Di Paola et al. [1].

### 2.3 LC-DAD-MS analysis

The phenolic compounds from tomato samples were analyzed by HPLC-DAD-ESI-MS/MS method. The equipment and procedure are described in Di Paola et al. [1]. Tentative identification of phenolic compounds was based on their retention times, elution order, UV–vis spectra and MS fragmentation spectra as compared with phenolic standards (Table 2). For polyphenols quantification, the mass peak areas were obtained from the extracted ion chromatograms (Fig. 2, Fig. 3).

### 2.4 Relationship between antioxidant composition and fruits traits

Principal component analysis (PCA) of antioxidant composition and fruit traits of tomato collection was performed using Infostat Software [2]. Fig. 3 shows the biplot graphic of two main principal components: (a) shows the score plot displaying fruit traits and (b) shows the loading plot displaying the antioxidant metabolites.

### 2.5 Relationship between antioxidant composition and geographical origin

PCA was also performed to access the correlations between antioxidant composition and geographical origin (and altitude). Biplot graphics of the two main components are shown in Fig. 4: (a) shows the score plot of geographical origin and altitude, and (b) shows the loading plot of antioxidant metabolites.

### 2.6 *C. elegans* assay

For in vivo assays, 1 mL of hydroalcoholic extract from each of the three biological replicates was pooled and dried. Afterwards, the dry residue was dissolved in 200 µl dimethyl sulfoxide (DMSO) and kept at −80 °C until use.

Worms were reproduced and maintained at 20 °C on nematode growth medium (NGM) plates using *Escherichia coli* OP50 as food source. When worms reached the young adult stage (three days after synchronization of the worms [3]), at least 30 young adults were transferred to plates containing fresh medium supplemented with serial dilutions of the tomato fruit extracts. Each experiment was carried out in triplicate using the hydroalcoholic extracts and 1% (v/v) DMSO as a vehicle control. After incubation at 20 °C for 18 h, worms were subjected to heat stress by treatment at 37 °C for 5 h 40 min causing the death of more than 50% of the population incubated in DMSO control. Worms were scored as dead when they failed to move in response to touch with a platinum wire.

Survival rate was dependent on the extract dilution and in most accessions an inverted *U* curve was obtained, which, on the one hand, it is produced by a toxic effect of the extract at high concentrations and, on the other, by the thermotolerance activity; therefore, the doses–response curve (log µg tomato vs. % worms rescued from death with respect to control) with extract dilutions, showing no toxic effects, was plotted for each accession (Fig. 5). The percentage of living (rescued) worms was calculated as rate of death worm in treatment×100/rate of death worm in control. Thermotolerance activity was expressed as the effective doses that produce 50% of rescued worms (ED50).

### 2.7 Relationship among in vivo and in vitro activities and fruit traits

To find relationships among in vivo and in vitro activities and fruit traits, hierarchical cluster analysis and PCA were performed using Infostat Software [2]. Dendrogram of fruit traits obtained by complete linkage clustering method is shown in Fig. 6a. Biplot graphic of the first two principal components shows the score plot of fruit traits and the loading plot of in vitro and in vivo activities (Fig. 6b).

### 2.8 Relationship among in vivo and in vitro activities and geographical origin

Fig. 7 shows the hierarchical cluster analysis and PCA, relating in vivo and in vitro activities to geographical origin (and altitude) of the tomato collection. Dendrogram of geographical origin obtained by complete linkage clustering method is shown in Fig. 7a. Biplot graphic of the first two principal components shows the score plot of geographical origin and the loading plot of in vitro and in vivo activities (Fig. 7b).

## Figures and Tables

**Table 1:** Wild and cultivated species; INTA La Consulta Horticulture Germplasm accession number; country, province and locality of accessions; tomato fruit characteristics and uses. Landraces accessions (L), commercial accessions (C) and wild tomato accessions (W).

Species	Germplasm accession no.	Country, province and locality	Fruit characteristics	Uses
*Solanum lycopersicum*	3819 (L)	Argentina, La Plata	Red, heart shape, slightly segmented, multilocular (8 cm diameter – 180 g)	Fresh consumption
*Solanum lycopersicum*	565 (L)	Argentina, Jujuy, Patacal	Red, slightly flattened, and segmented multilocular (6,5 cm diameter – 120 g)	Fresh consumption
*Solanum lycopersicum*	3842 (L)	Argentina, Mendoza, Las Heras	Red, flattened, slightly segmented, multilocular (8–9 cm diameter – 200 g)	Fresh consumption
*Solanum lycopersicum*	4735 (C)	Commercial is a local selection of M-82	Red, elliptical, uniform, 2–3 locules (4.5–5 cm diameter – 70 g)	Processing
*Solanum lycopersicum*	3815 (L)	Argentina, Mendoza, Luján	Red cherry, globose, uniform, 2 locules (1.8 cm diameter – 3 g)	Fresh consumption
*Solanum lycopersicum*	571 (L)	Argentina, Catamarca, Santa María	Red, elliptical-globular, slightly flattened and segmented, 2–3 locules (5 cm diameter – 82 g)	Fresh consumption
*Solanum lycopersicum*	GPEA (Garden Peach) (C)	Commercial	Pale yellow cherry, globose, uniform, 2–3 locules (2.5 cm diameter – 8 g)	Fresh consumption
*Solanum lycopersicum*	572 (L)	Argentina, Catamarca, Santa María	Red, elliptical, uniform, 2 locules (2 cm diameter – 6 g)	Fresh consumption
*Solanum lycopersicum*	3806 (L)	Argentina, Mendoza, Luján is a local selection from seeds of a San Marzano variety, originally from Italy	Red, elongated, 2–3 locules (4–5 cm diameter, 10–12 cm length, 100 g)	Fresh consumption and processing
*Solanum lycopersicum*	560 (L)	Argentina, Salta, Luracatao	Yellow, flattened and segmented, multilocular (7 cm diameter – 110 g)	Fresh consumption
*Solanum habrochaites*	LA407 (collection SAL 160) (W)	Ecuador, Guayas, Guayaquil, El Mirador	Green with two dark strips, slightly flattened, 2 locular (1.5 cm diameter – 2.5 g)	Not edible
*Solanum lycopersicum*	4742 (C) Yellow Stuffer	Commercial	Yellow, pepper shape, 2–3 loculars, unfilled locule (6 cm diameter – 70 g)	Fresh consumption
*Solanum lycopersicum*	552 (L)	Argentina, Catamarca Hulafín	Red, elliptical, uniform, 2–3 locules (4.5–5 cm diameter – 70 g)	Fresh consumption and processing
*Solanum lycopersicum*	4750 (L)	Argentina, Luján, Mendoza	Purplish-blue cherry, globose, uniform, 2 locules (2.5 cm diameter – 6 g)	Fresh consumption
*Solanum lycopersicum*	557 (L)	Argentina, Salta, Luracatao	Red, pear shape, slightly segmented, multilocular (6–7 cm diameter – 120 g)	Fresh consumption
*Solanum pimpinellifolium*	4739 (W) (LA1589, collection SAL 1869)	Peru, La Libertad, Viru Galunga	Red currant, globose, uniform, 2 locules (1 cm diameter – 1 g)	Strong flavor edible

**Table 2:** Chromatographic characteristics, UV–visible and mass spectral data of phenolic compounds identified in tomato accessions.

Peak	tR (min)	Molecular ion [M–H]^−^ (*m*/*z*)	MS–MS of [M–H]− (*m*/*z*)	*λ* (max) (nm)	Tentative identification
1	6.7	341	179, 161	n.d.	Caffeic acid-O-hexose I
2	7.2	353	191, 179	325	Chlorogenic acid isomer
3	7.3	371	209, 191	n.d.	Caffeoyl hexaric acid
4	11.6	353	191, 179	325	Neochlorogenic acid
5	11.9	341	179, 161, 135	287, sh 303	Caffeic acid-O-hexose II
6	11.9	325	163	287	Coumaric acid-O-hexose I
7	12	371	191	n.d.	Trihydroxy cinnamoylquinic acid
8	12.1	355	193	313	Ferulic acid-O-hexoside
9	12.4	341	179	n.d.	Caffeic acid-O-hexose III
10	12.4	353	191, 179	325, sh 294	Chlorogenic acid
11	12.7	341	179.135	n.d.	Caffeic acid-hexose IV
12	12.8	325	163	286	Coumaric acid-hexose II
13	13.3	353	191	325	Cryptochlorogenic acid
14	13.7	341	n.d.	n.d.	Caffeic acid-hexose V
15	18.0	609	301	354	Rutin
16	24.1	271	151	366	Naringenin-chalcone
